# Accelerating the Radiology Learning Curve: How the Parameter-Based I-TIRADS Lexicon Enhances Interpretive Reliability and Learning Curves Across Resident Experience Levels—A Dynamic Video-Based Study

**DOI:** 10.3390/diagnostics16193103

**Published:** 2026-09-24

**Authors:** Burak Yangoz, Mustafa Sagan, Ismet Duman, Ahmet Sukru Alparslan, Tunc Burak Benkaya, Sergen Palaz, Mustafa Berk Silbir, Ender Uysal

**Affiliations:** Antalya Training and Research Hospital, University of Health Sciences, Antalya 07100, Turkey; saganmustafa@gmail.com (M.S.); ismetduman92@hotmail.com (I.D.); ahmetalprsln@gmail.com (A.S.A.); benkayab@gmail.com (T.B.B.); sergen.palaz07@gmail.com (S.P.); berk_1997_61@hotmail.com (M.B.S.); enderun73@gmail.com (E.U.)

**Keywords:** I-TIRADS, radiology education, diagnostic consistency, thyroid nodules, ultrasonography

## Abstract

**Background/Objectives:** Standardizing thyroid nodule assessment remains a major challenge in clinical practice and radiology education. Traditional categorical risk stratification systems (RSSs) often yield high interobserver variability, particularly among trainees. This study evaluated the reproducibility, educational utility, and experience-dependent performance of the parameter-based International Thyroid Imaging Reporting and Data System (I-TIRADS) lexicon compared to established categorical RSSs (ACR, EU, and K-TIRADS). **Methods:** In this prospective validation study, 105 thyroid nodules stratified across Bethesda categories were evaluated by seven radiologists: one senior expert (as reference standard) and six residents categorized into three distinct experience levels (Junior, Intermediate, Senior). To simulate real-time clinical practice and eliminate 2D static image bias, comprehensive dynamic video clips were utilized for all cases. Interobserver agreement and intergroup consistency were quantified using Fleiss’ and Cohen’s kappa (*κ*) statistics. **Results:** Parameter-based I-TIRADS descriptors—specifically composition (*κ* = 0.510) and direction of growth (*κ* = 0.498)—demonstrated significantly higher overall reliability compared to final categorical RSS classifications (ACR: *κ* = 0.220, EU: *κ* = 0.215, K-TIRADS: *κ* = 0.196). Crucially, the reproducibility advantage of the I-TIRADS lexicon became markedly more pronounced with advancing clinical experience: senior observers achieved substantial to near-perfect consensus for core descriptors (up to *κ* = 0.826), whereas agreement for traditional RSS categories remained limited. I-TIRADS facilitated a steeper learning curve, allowing even junior trainees to achieve reliable consensus on objective sonographic parameters. **Conclusions:** The parameter-based I-TIRADS provides superior interobserver consistency compared to traditional categorical RSSs by reducing subjective evaluative drift, accelerating the radiologic learning curve, and offering a highly reproducible framework for both clinical practice and residency education.

## 1. Introduction

Thyroid nodules are a common clinical finding in adult populations, with detection rates reaching up to 67% when high-resolution ultrasonography (US) is utilized [1]. Despite the high sensitivity of US in detecting nodules, substantial interobserver variability remains a significant challenge in the standardized reporting and risk stratification of these lesions [2,3]. This variability often stems from the subjective interpretation of sonographic descriptors, which can lead to disparate management recommendations for the same nodule. To overcome these inconsistencies, several RSSs, such as American College of Radiology (ACR)-TIRADS, European Thyroid Association (EU)-TIRADS, and Korean (K)-TIRADS, have been developed to provide a standardized lexicon and objective malignancy risk assessment [4,5,6]. Previous studies have indicated that the diagnostic performance and interobserver consistency of these systems are often influenced by the level of experience of the interpreting radiologist, suggesting that junior radiologists may struggle with the nuanced definitions within these frameworks [7,8].

The I-TIRADS was proposed as a standardized lexicon to simplify US reporting and enhance interobserver consistency, although it does not yet function as a complete risk stratification scoring system [9]. While recent preliminary studies, such as Uysal et al., have evaluated the general interobserver reproducibility of individual I-TIRADS descriptors using dynamic ultrasound recordings, significant knowledge gaps remain regarding how trainee experience levels modulate descriptor interpretation and how I-TIRADS compares directly against established categorical RSSs (ACR, EU, and K-TIRADS) in an educational setting. Furthermore, no prior study has investigated how the parameter-based I-TIRADS lexicon impacts the radiologic learning curve across distinct residency training stages. This study addresses these critical limitations by providing a head-to-head prospective comparison of the I-TIRADS lexicon versus three major categorical RSSs across three stratified resident experience tiers (Junior, Intermediate, and Senior), evaluated via standardized dynamic video clips. Rather than asserting diagnostic accuracy superiority in a limited cohort, our primary objective is to evaluate interobserver agreement, diagnostic consistency, and the educational acceleration enabled by parameter-based reporting in radiology residency [2,10].

Therefore, the present study aims to evaluate the impact of the I-TIRADS lexicon on interobserver agreement and diagnostic consistency across different levels of radiologic experience, while providing a head-to-head comparison with the ACR, EU, and K-TIRADS systems [4,5,9,11].

## 2. Materials and Methods

### 2.1. Study Design and Ethics

This prospective validation study was conducted at the University of Health Sciences, Antalya Training and Research Hospital. The study protocol was approved by the Clinical Research Ethics Committee of Antalya Training and Research Hospital (Date: 26 September 2024; Decision No: 14/16). The study was performed in accordance with the Declaration of Helsinki.

### 2.2. Patient Selection and Nodule Population

Between 1 January and 1 October 2024, a total of 600 consecutive patients scheduled for thyroid nodule fine-needle aspiration biopsy (FNAB) were screened. To ensure a high-quality dataset, cases with inadequate cytopathological sampling or suboptimal image quality were excluded. From the remaining eligible pool, 105 patients (89 females, 16 males; mean age 51.1 ± 13.2 years) were selected using a stratified random sampling technique based on Bethesda System for Reporting Thyroid Cytopathology (BSRTC)categories to ensure a balanced representation of both benign and malignant cases across the study cohort [12,13]. This process ensured the study population mirrored the epidemiological distribution of BSRTC categories (I–VI) in the general population, thereby minimizing selection bias. For patients with multiple nodules, only the specifically sampled nodule was evaluated to maintain statistical independence.

### 2.3. Observers and Image Evaluation

Seven radiologists independently evaluated the cases. A senior radiologist (>15 years of specialized experience in thyroid US) served as the senior expert reference standard for US descriptor baseline comparison. Importantly, because all resident observers received their formal ultrasound training within the same academic department under the supervision of this senior faculty member, this setup specifically enabled us to evaluate mentor-mentee concordance, institutional learning curve dynamics, and experience-dependent evaluative drift across residency training stages [14,15]. The other six observers were categorized into three experience-based groups: Junior (1st year of residency), Intermediate (2nd year), and Senior (3rd year), with two observers in each group to minimize individual bias through mean value analysis. Regarding the effect of the pre-evaluation orientation session, all resident observers completed a standardized 1-h orientation prior to image evaluation in order to standardize the application of the lexicon and the RSSs [15]. All observers were blinded to clinical data, pathology results, and the evaluations of the other participants.

### 2.4. US Assessment and RSSs Application

All ultrasonographic examinations were performed using a Toshiba Aplio 500 (Canon Medical Systems, Tokyo, Japan) US system equipped with a high-frequency 7.5 MHz linear array transducer. For each patient, high-resolution axial, sagittal, and coronal static images of the biopsied nodule were recorded at its largest dimensions. To eliminate 2D static image selection bias and mimic real-time point-of-care clinical examinations, standardized high-definition dynamic video clips were systematically recorded for all 105 thyroid nodules [16]. To control for operator-dependent acquisition bias, all examinations were performed by a single senior radiologist using a uniform sweep protocol. The transducer was systematically swept across the entire nodule volume in both transverse and longitudinal planes, including the surrounding parenchyma and cervical lymph node compartments (Levels I–VI). Ultrasound system parameters—including gain, focal depth, dynamic range, and time gain compensation—were optimized and kept constant across all sessions to maintain uniform spatial and contrast resolution. Video clips were captured at 30 frames per second with a mean duration of 32 ± 8 s (range: 20–45 s) per lesion. All anonymized recordings were hosted on a secure digital server, granting evaluators unlimited playback, speed modulation, and frame-by-frame analysis without time constraints. Representative ultrasonographic images of the thyroid nodules included in the study, showcasing various I-TIRADS descriptors and risk categories, are presented in Figure 1 and Figure 2.

All media were fully anonymized by removing identification data before the evaluation phase. The observers characterized each nodule based on I-TIRADS descriptors (composition, echogenicity, margin, direction of growth, echogenic foci/calcifications, extrathyroidal extension (ETE), and lymph node status) and simultaneously classified them according to ACR TI-RADS, EU-TIRADS, and K-TIRADS guidelines [4,5,6,9]. No external resources were accessible to the observers during this process. The quality of the recorded media was verified by the senior expert radiologist prior to the evaluation phase.

### 2.5. Statistical Analyses

Statistical analyses were performed using SPSS version 27.0 (IBM Inc., Chicago, IL, USA). The sample size calculation was predicated on the 5-category ordinal scale of ACR TI-RADS (TR1–TR5), which represents the most statistically demanding parameter in our dataset, carrying the highest degrees of freedom. Establishing sufficient statistical power for this 5-level categorical scale ensures that all secondary binary or 3-level descriptors in the dataset are inherently adequately powered. Assuming a null hypothesis of fair agreement (*κ* ≤ 0.40) against an alternative hypothesis of substantial agreement (*κ* = 0.70), with a two-tailed alpha of 0.05 and a sample size of 105 thyroid nodules, the study achieved adequate statistical power (>80%) to evaluate interobserver agreement across both multiclass categories and individual lexicon parameters without Type II error. Descriptive statistics were presented as mean ± standard deviation for normally distributed variables and as median (minimum–maximum) for non-normal data. The normality of distribution was assessed using the Kolmogorov–Smirnov test and histogram analysis. Interobserver agreement across the experience groups and against the reference standard was evaluated using Fleiss’ and Cohen’s kappa (*κ*) statistics, respectively. The strength of agreement was interpreted according to Cohen’s criteria: <0.20, slight; 0.21–0.40, fair; 0.41–0.60, moderate; 0.61–0.80, substantial; and 0.81–1.00, almost perfect agreement. A *p*-value of <0.05 was considered statistically significant.

## 3. Results

### 3.1. Baseline Patient and Nodule Characteristics

The study included a total of 105 patients, with a mean age of 51.1 ± 13.2 years (range: 19–78). There was a significant female predominance, accounting for 84.8% (*n* = 89) of the cohort. Regarding nodule size, the majority of the lesions were ≥1 cm (93.3%, *n* = 98), with a median diameter of 18 mm (range: 7–62 mm). Cytopathological evaluation based on the BSRTC System revealed that 61% (*n* = 64) of the nodules were benign (Bethesda II), while 7.6% (*n* = 8) were malignant (Bethesda VI). The stratified random sampling ensured a balanced distribution across BSRTC categories III, IV, and V, representing 29.5%, 1%, and 1% of the cases, respectively. Detailed demographic data and nodule characteristics are presented in Table 1.

### 3.2. Interobserver Agreement on I-TIRADS Lexicon Parameters

The interobserver agreement for the sonographic parameters defined in the I-TIRADS lexicon, as well as the overall categories of established RSSs, was evaluated using Fleiss’ *κ* analysis (Table 2). Among all the observers, the highest levels of consensus were achieved for composition (*κ* = 0.510) and direction of growth (*κ* = 0.498). Parameters such as echogenicity (*κ* = 0.385) and echogenic foci/calcifications (*κ* = 0.382) demonstrated moderate levels of agreement. Relatively lower levels of consistency were observed for margin characteristics (*κ* = 0.254), ETE (*κ* = 0.273), and lymph node assessment (*κ* = 0.286). Notably, when compared to the individual descriptors of the I-TIRADS lexicon, the overall agreement for the established risk stratification systems was significantly lower. Specifically, ACR-TIRADS (*κ* = 0.220), EU-TIRADS (*κ* = 0.215), and K-TIRADS (*κ* = 0.196) demonstrated limited interobserver consistency, highlighting that standardized lexicon descriptors may offer more reliable consensus than the final categorical classifications of current RSSs.

### 3.3. Impact of Clinical Experience on Interobserver Agreement

The analysis of interobserver agreement across different experience levels revealed a direct correlation between clinical seniority and diagnostic consistency. As illustrated in the line graph (Figure 3), there was a steady upward trend in *κ* values for all I-TIRADS lexicon parameters as years of experience increased.

The highest level of consensus was achieved among the Senior residents and the senior expert (Table 3). For core I-TIRADS descriptors, the agreement reached substantial to almost perfect levels, particularly for direction of growth (*κ* = 0.826, 96.8% agreement), echogenic foci/calcifications (*κ* = 0.808), composition (*κ* = 0.743), and margin (*κ* = 0.701). Despite this high performance in lexicon parameters, the agreement for established RSSs remained significantly lower, with ACR-TIRADS, EU-TIRADS, and K-TIRADS yielding kappa values of 0.466, 0.368, and 0.273, respectively.

In the Intermediate group, moderate agreement was maintained for direction of growth (*κ* = 0.491), composition (*κ* = 0.464), and echogenicity (*κ* = 0.429), while consistency dropped for ETE (*κ* = 0.185) and lymph nodes (*κ* = 0.227) (Table 4). Among Junior observers, the agreement was generally limited to fair or slight levels. While direction of growth still provided the highest consensus in this group (*κ* = 0.431), most other parameters such as echogenicity (*κ* = 0.220) and lymph nodes (*κ* = 0.118) showed very low consistency. Notably, the evaluation of nodule margins failed to reach statistical significance among Junior observers (*κ* = 0.076, *p* = 0.078), highlighting that margin assessment represents a significant learning curve in thyroid US (Table 5) [13,14]. Across all experience levels, the parameter-based evaluation of the I-TIRADS lexicon consistently outperformed the categorical scoring of traditional RSSs.

### 3.4. In-Depth Subgroup Analysis of I-TIRADS and RSS Parameters

To further explore the nuances of interobserver consistency, a subgroup analysis was performed across different experience levels. Detailed results for each experience group (Junior, Intermediate, and Senior) are provided in the Supplementary Tables S2–S4.

Across all the cohorts, the highest and most stable levels of agreement were consistently observed for spongiform and solid compositions, as well as the taller-than-wide orientation. In the Senior group, these features reached near-perfect consensus (*κ* up to 0.897), while even Junior observers maintained their highest relative scores in these subcategories. Similarly, comet-tail artifacts and the absence of calcifications stood out as the most reliably identified echogenic foci across all experience levels.

The analysis identified specific “subjective hurdles” where agreement remained low regardless of experience. Specifically, suspected minor ETE and indeterminate lymph nodes yielded the lowest kappa values across all three groups. A significant learning curve was evident in margin assessment and echogenicity (specifically hyperechoic and anechoic patterns), where Junior and Intermediate observers struggled to reach meaningful consensus, whereas Senior observers achieved substantial agreement.

In contrast to the high reliability of individual I-TIRADS descriptors, the specific categories of ACR-TIRADS, EU-TIRADS, and K-TIRADS showed poor interobserver reliability. Consistency was particularly low for intermediate-risk categories (Categories 2, 3, and 4) across all systems, with many failing to reach statistical significance among Junior and Intermediate evaluators. This highlights that while individual lexicon terms are becoming standardized, their collective integration into final RSS categories remains highly subjective.

## 4. Discussion

### 4.1. Comparative Analysis of Lexicon-Based Assessment and Categorical RSSs and Methodological Dynamics

A principal finding of our study is that individual descriptors defined in the parameter-based I-TIRADS lexicon yield significantly higher interobserver reproducibility compared to the overall categorical classifications of established RSSs. Our analysis revealed limited consensus for traditional RSSs, with Fleiss’ *κ* values of 0.220 for ACR-TIRADS, 0.215 for EU-TIRADS, and 0.196 for K-TIRADS across all observers. In contrast, core I-TIRADS lexicon parameters—specifically composition (*κ* = 0.510) and direction of growth (*κ* = 0.498)—demonstrated moderate to substantial agreement.

This discrepancy stems from the fundamental structural difference between parameter-based reporting and categorical risk scoring, as well as the disparate weighting assigned to identical sonographic features across established RSSs [17]. In ACR-TIRADS, risk is determined via an additive point-summation model across five categories, where minor subjective disagreements accumulate cumulatively [4]. Conversely, EU-TIRADS operates on a pattern-based threshold model where a single cardinal high-risk feature—such as marked hypoechogenicity or irregular margins—automatically upgrades a nodule to EU-TIRADS 5 (High Risk) [5]. K-TIRADS uses a hybrid approach, relying on a solid-hypoechoic baseline modified by counting three specific suspicious features [6]. Consequently, identical descriptor observations propagate differently across systems. For example, a subjective distinction between mild and marked hypoechogenicity directly shifts a nodule from EU-TIRADS 4 to EU-TIRADS 5, adds one point in ACR-TIRADS (potentially crossing TR3 to TR4 or TR4 to TR5 thresholds), but maintains the same baseline tier in K-TIRADS unless secondary counting features are present. Similarly, ACR-TIRADS allows cumulative scoring for concurrent calcification patterns (e.g., punctate foci plus macrocalcifications), whereas EU-TIRADS and K-TIRADS evaluate microcalcifications as a discrete binary high-risk trigger. These structural variations explain why trainees who achieve acceptable consensus on individual I-TIRADS parameters nevertheless assign divergent final risk categories.

Although biopsy-based cohorts can inherently introduce selection bias, this was effectively mitigated in our study by employing stratified random sampling based on Bethesda categories to mirror their epidemiological distribution in the general population, thereby establishing a representative real-world series [12,13]. Given our sample size of 105 thyroid nodules and a limited number of histologically confirmed malignant cases (*n* = 8, 7.6%), it is essential to clarify that this study was intentionally not designed or statistically powered to evaluate diagnostic accuracy or performance metrics such as sensitivity, specificity, or ROC AUC (Receiver Operating Characteristic Area Under the Curve) superiority. Instead, our investigation was strictly structured to evaluate interobserver reproducibility, agreement stability across training levels, and the mitigation of evaluative drift among trainees.

Traditional RSSs integrate individual sonographic features into aggregated risk categories via point-summation or pattern-matching algorithms [4,5,6]. Consequently, minor subjective disagreements on a single feature (e.g., mild versus marked hypoechogenicity) propagate exponentially, shifting a nodule across categorical risk thresholds and causing substantial evaluative drift among trainees [18]. By decoupling objective descriptor identification from rigid category assignment, the I-TIRADS lexicon minimizes these evaluative shifts [9]. Even though radiologists generally achieve acceptable agreement on discrete sonographic features, establishing consensus on aggregated categorical risk tiers remains inherently challenging [19]. Prior assessment training on I-TIRADS parameters, despite enabling observers to reach consensus on clearly defined features such as growth direction and composition, remains inherently limited in scope. Brief orientation sessions are unable to resolve subjective differences regarding sophisticated or subtle features, such as irregular margins or mild hypoechogenicity. Thus, to address this gap, junior residents require continuous, long-term feedback combined with case studies linked to pathological findings [15].

Because all resident raters received formal ultrasound training within the same academic department under the direct supervision of the reference faculty member, shared departmental diagnostic habits likely contributed to a higher baseline mentor–mentee concordance. In addition, unlike many studies relying solely on static images, our methodology incorporated dynamic video clips for each nodule to better simulate real-time clinical assessment and mitigate the inherent limitations of 2D imaging [20]. Despite these optimized visualization conditions and shared institutional co-training, the agreement remained “slight” to “fair” across established RSSs. Our findings reported lower *κ* values (0.196–0.220) compared to those of Seifert et al. (0.321–0.411), a discrepancy that warrants a multi-faceted interpretation [20]. We attribute this primarily to the hierarchical gap in observer experience; while previous literature often utilizes expert-level readers, our cohort consisted of junior and intermediate residents. This underscores that even with comprehensive dynamic imaging, the subjective weighting of nuanced features—such as the distinction between “hypoechoic” and “markedly hypoechoic”—remains highly susceptible to interobserver drift among trainees [2,14,15,16]. This diversity naturally introduces greater variability, highlighting that RSSs are particularly sensitive to the observer’s level of training, as subjectivity in weighting different features often leads to “interobserver drift” [15,21].

In traditional systems, categorical definitions are often susceptible to varied sonographic interpretations of imaging features, leading to lower agreement levels that can undermine diagnostic confidence and complicate the clinical decision-making process [2,15,18]. By providing a structured, simplified, and standardized sonographic language, I-TIRADS minimizes these evaluative discrepancies. This effort to establish a universal US language, as advocated by Durante et al., represents a promising approach for both academic research and daily clinical practice [9].

On the other hand, beyond the intranodular sonographic descriptors evaluated in the current I-TIRADS lexicon, emerging evidence emphasizes the diagnostic importance of the tumor microenvironment and peritumoral tissue zone. Recent studies demonstrate that quantitative features extracted from the immediate peritumoral periphery capture critical biological signals—such as peritumoral stromal reactions and microvascular changes—that significantly outperform or complement traditional intranodular analysis alone in lesion discrimination [22]. While current ultrasound guidelines address frank ETE or capsular contact, subtle peritumoral parenchymal alterations remain unquantified in visual reporting. Integrating standardized peritumoral zone descriptors into future lexicon iterations, particularly when paired with artificial intelligence algorithms, could offer richer diagnostic features and further refine observer agreement [22].

### 4.2. Impact of Clinical Experience and the Learning Curve

Our analysis reveals a direct correlation between clinical seniority and interobserver consistency, aligning with previous findings in thyroid ultrasound education [15,21]. We observed a significant upward trend in *κ* values across almost all I-TIRADS lexicon parameters as years of experience increased (Figure 3). Notably, while I-TIRADS showed a substantial improvement in agreement with experience, the consensus for categorical RSSs remained relatively stagnant or showed only marginal gains. This suggests that the I-TIRADS lexicon is not merely a reporting tool but also a highly learnable framework that supports a progressive learning curve [9].

To statistically validate this experience-dependent progression, we examined the overlap dynamics of the 95% CIs across resident tiers. In statistical reliability frameworks, non-overlapping 95% CIs between independent cohorts denote statistically significant differences in concordance. Notably, subjective parameters that initially posed severe interpretive obstacles for Junior residents showed entirely non-overlapping CIs when compared to Senior trainees, confirming genuine learning curve progression rather than random variation. For instance, in nodule margin evaluation, Junior residents achieved only slight agreement (*κ* = 0.076, 95% CI: −0.009 to 0.160), whereas Senior residents reached substantial agreement (*κ* = 0.701, 95% CI: 0.609 to 0.792), exhibiting completely distinct intervals. Similarly, for nodule composition and echogenic foci/calcifications, Senior residents (*κ* = 0.743, 95% CI: 0.670 to 0.817, *κ* = 0.808, 95% CI: 0.742 to 0.874, respectively) demonstrated a marked superiority over Junior trainees (*κ* = 0.403, 95% CI: 0.325 to 0.481, *κ* = 0.252, 95% CI: 0.183 to 0.320, respectively). Even for robust parameters such as growth direction, the Senior cohort (*κ* = 0.826, 95% CI: 0.716 to 0.937) significantly outperformed the Junior group (*κ* = 0.431, 95% CI: 0.321 to 0.542) without interval overlap. Conversely, categorical risk stratification systems failed to exhibit such distinct CI separation across experience tiers, reinforcing the concept that parameter-based reporting uniquely accelerates skill acquisition for challenging sonographic descriptors.

A critical finding of this study is that the level of subjectivity inherent in certain sonographic features acts as a primary hurdle for novice evaluators [21]. Among Junior observers, the evaluation of nodule margins failed to reach statistical significance (*κ* = 0.076, *p* = 0.078), and agreement for ETE (*κ* = 0.194) and lymph node assessment (*κ* = 0.118) remained poor. Unlike parameters such as direction of growth or size—which are based on more objective geometric measurements—margin, ETE, and lymph node assessments rely heavily on qualitative visual interpretation. As noted by McQueen and Bhatia, the spatial resolution in thyroid US is asymmetric; while axial resolution is highly precise due to high-frequency transducers, lateral resolution is consistently inferior as it is limited by the ultrasound beam width [18,23]. This technical constraint often leads to “lateral blurring” at the interface between the nodule and the surrounding tissue, complicating the qualitative assessment of margins and borders. This highlights that features requiring nuanced visual perception are the most difficult to standardize in early residency [10,14,21]. In contrast, descriptors that are more objective or visually distinct—such as direction of growth and composition—maintained moderate levels of agreement even among Junior observers (*κ* = 0.431 and *κ* = 0.403, respectively). These results echo the findings of Koh et al., who emphasized that binary or measured features are less susceptible to interpretive variance [21].

As noted by Durante et al., deconstructing nodule assessment into discrete, well-defined parameters may facilitate a more structured learning process relative to the integrative categorical assessment characteristic of traditional RSSs [9]. This consistency is vital to reduce diagnostic uncertainty and prevent unnecessary FNAB’s [24]. The high agreement in the I-TIRADS lexicon may suggest it can serve as a “universal language” for training artificial intelligence (AI) algorithms, ensuring that the “ground truth” used for machine learning is consistent [25,26]. For residency programs, our results suggest that training should focus heavily on subjective parameters such as “margin” and “echogenicity”, while leveraging the high reliability of “direction of growth” to build diagnostic confidence [10].

### 4.3. Limitations

Despite its strengths, this study has limitations. First, it was conducted at a single tertiary center, which may limit the generalizability of the results to different clinical settings. Second, the number of malignant nodules was small (*n* = 8, 7.6%); therefore, all the findings in this study must be interpreted strictly in terms of interobserver agreement and educational learning curve acceleration rather than diagnostic efficacy. Future prospective multicenter studies with substantially larger malignant cohorts are required to establish whether the observed interobserver consistency translates into improved diagnostic accuracy. Third, a single senior expert radiologist served as the reference baseline for descriptor comparison. This approach was taken to evaluate mentor–resident compatibility and the dynamics of the educational curve among residents trained under a uniform curriculum. To validate these training dynamics, conducting multicenter training studies involving multi-reader expert panels in the future is recommended.

## 5. Conclusions

Our study demonstrates that the parameter-based I-TIRADS lexicon provides significantly higher interobserver agreement compared to traditional categorical RSSs, effectively reducing subjective evaluative drift and facilitating a steeper learning curve for trainees across all experience levels.

## Figures and Tables

**Figure 1:**
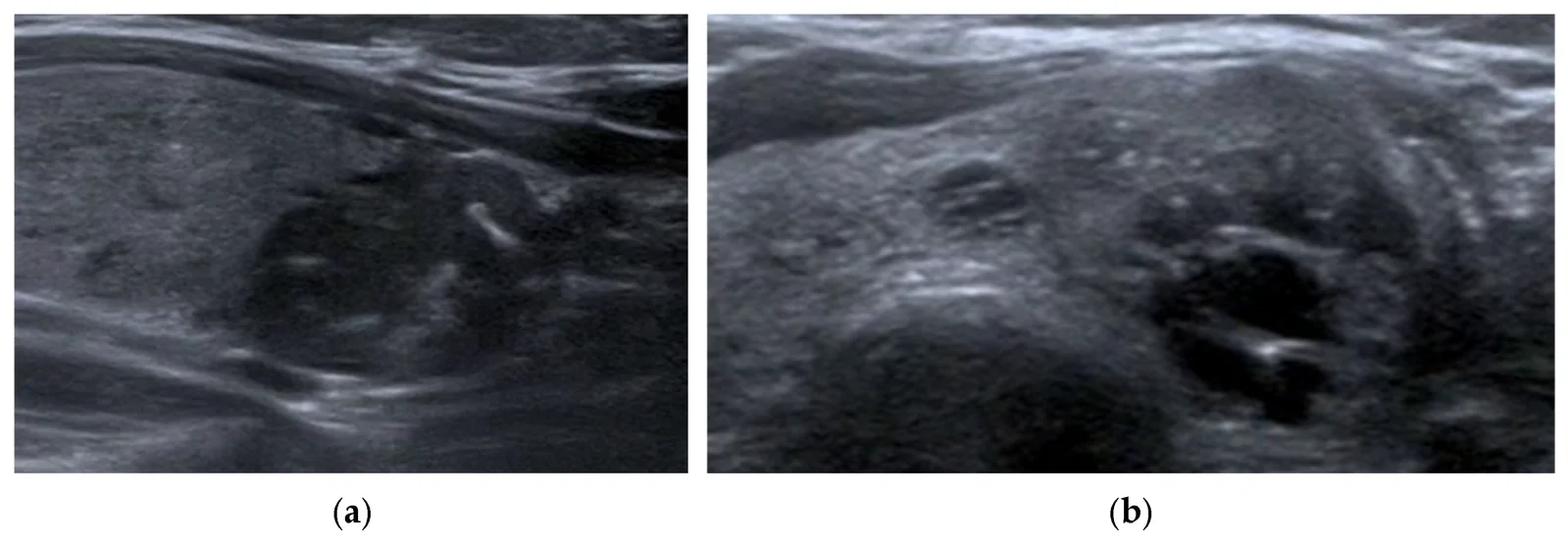
Representative ultrasonographic features of high-risk and malignant thyroid nodules: (**a**) Coronal ultrasound section of the left thyroid lobe revealing a markedly hypoechoic nodule localized in the inferior aspect of the gland with internal macrocalcifications, consistent with high-risk features defined by the International Thyroid Imaging Reporting and Data System I-TIRADS lexicon (subsequent fine-needle aspiration biopsy resulted in Bethesda System for Reporting Thyroid Cytopathology BSRTC Category V, Suspicious for Malignancy); (**b**) Transverse ultrasonography (US) section of the left thyroid lobe demonstrating a mixed, predominantly solid, hypoechoic nodule with lobulated margins, internal microcalcifications, and indentation of the anterior strap muscles indicating suspected extrathyroidal extension (ETE) (postoperative histopathological examination confirmed Papillary Thyroid Carcinoma).

**Figure 2:**
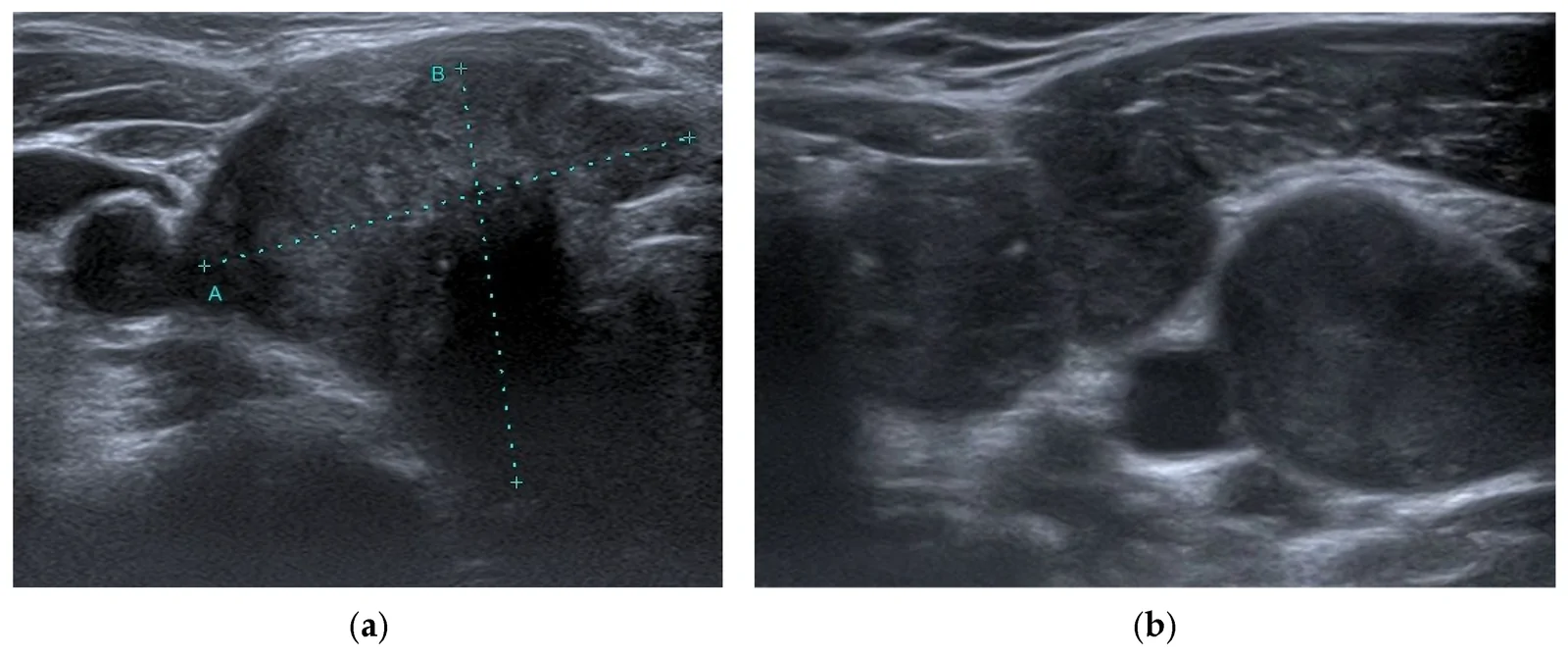
Ultrasonographic spectrum of Papillary Thyroid Carcinoma (PTC) presenting with vertical orientation and metastatic lymphadenopathy: (**a**) Transverse ultrasound image of the right thyroid bed showing a 33(A) × 28(B) mm nodule (where A and B denote the maximum orthogonal caliper measurements) with a taller-than-wide orientation, patchy markedly hypoechoic areas, and ill-defined posterior margins—features that often contribute to interobserver variability (postoperative histopathological examination confirmed PTC); (**b**) Axial ultrasound image revealing a large, hypoechoic nodule with lobulated margins involving the right thyroid lobe (on the left) and a corresponding pathological lymph node at the right jugular chain level (on the right), characterized by a rounded shape and loss of the normal fatty hilum (postoperative histopathological evaluation confirmed metastatic involvement of the regional lymph node).

**Figure 3:**
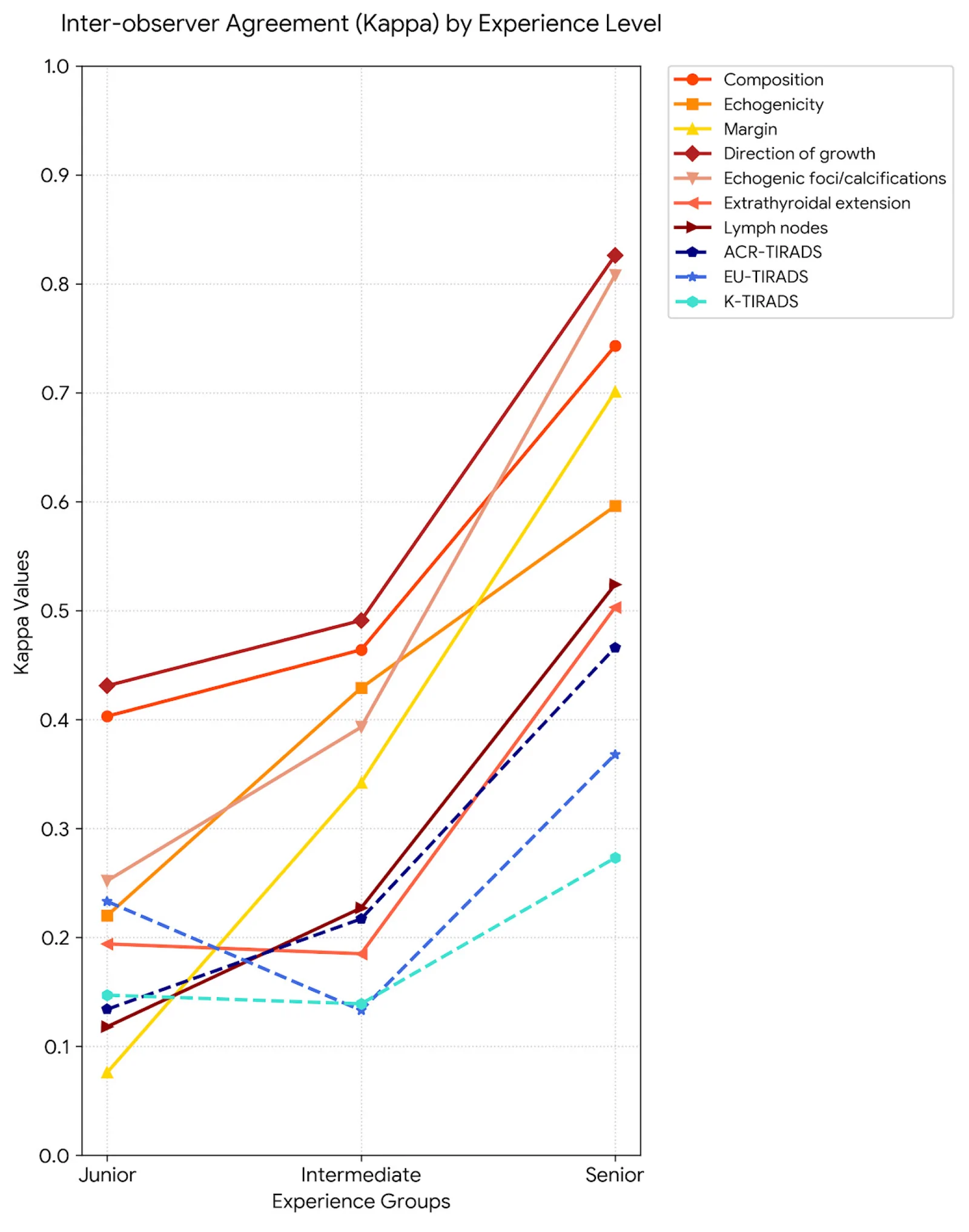
Comparative analysis of interobserver agreement (*κ*) for the International Thyroid Imaging Reporting and Data System (I-TIRADS) parameters and risk stratification systems (RSSs) based on experience levels. The line graph illustrates the evolution of interobserver consistency among observers with 1, 2, and 3 years of clinical experience. A consistent upward trend in *κ* values is observed across almost all parameters as experience increases. Notably, fundamental I-TIRADS lexicon descriptors achieved substantial to almost perfect agreement in the Senior group. In contrast, the agreement for established RSSs remained significantly lower, with values for American College of Radiology (ACR)-TIRADS, European Thyroid Association (EU)-TIRADS, and Korean (K)-TIRADS peaking at only *κ* 0.466, 0.368, and 0.273, respectively, even among the most experienced observers. (Note: Solid lines represent individual I-TIRADS lexicon parameters, while dashed lines represent established categorical risk stratification systems).

**Table 1:** Baseline clinicodemographic and ultrasonographic characteristics of the study population.

Parameters	Sex		*p*
	Male (*n* = 16, 15.2%)	Female (*n* = 89, 84.8%)	
	Distribution *		
Age (years)	58.56 ± 12.84	49.76 ± 12.9	0.013 ^a^
Nodule size (mm)	25.5 (10–62)	17 (7–50)	0.087 ^b^
Nodule multiplicity			
Solitary	0 (0%)	14 (15.73%)	0.121 ^c^
Multiple	16 (100%)	75 (84.27%)	
Nodule size distribution			
<1 cm	0 (0%)	7 (7.87%)	0.592 ^c^
≥1 cm	16 (100%)	82 (92.13%)	

* Parameters are expressed as quartiles (median, minimum, and maximum) or as the mean ± standard deviation (SD). ^a^ Mann-Whitney U test, ^b^ independent *t*-test, ^c^ Fisher’s exact test.

**Table 2:** Interobserver agreement levels of I-TIRADS parameters and established RSSs among all observers (Fleiss kappa analysis).

Parameters	*κ*	95% CI	*p*-Value *
Composition	0.510	0.481–0.539	<0.001
Echogenicity	0.385	0.357–0.413	<0.001
Margin	0.254	0.222–0.286	<0.001
Direction of growth	0.498	0.456–0.539	<0.001
Echogenic foci/calcifications	0.382	0.358–0.407	<0.001
Extrathyroidal extension	0.273	0.240–0.306	<0.001
Lymph nodes	0.286	0.253–0.319	<0.001
ACR-TIRADS	0.220	0.196–0.243	<0.001
EU-TIRADS	0.215	0.187–0.242	<0.001
K-TIRADS	0.196	0.172–0.221	<0.001

Abbreviations: CI = Confidence interval, *κ* = kappa value, ACR = American College of Radiology, EU = European Thyroid Association, K = Korean Thyroid Association. * Fleiss kappa analysis.

**Table 3:** Interobserver agreement for I-TIRADS parameters and established RSSs in the Senior group.

Parameters	Interobserver Agreement (%)	*κ*	95% CI	*p*-Value *
Composition	84.2%	0.743	0.670–0.817	<0.001
Echogenicity	73.2%	0.596	0.523–0.669	<0.001
Margin	86.7%	0.701	0.609–0.792	<0.001
Direction of growth	96.8%	0.826	0.716–0.937	<0.001
Echogenic foci/calcifications	86.7%	0.808	0.742–0.874	<0.001
Extrathyroidal extension	87.9%	0.503	0.412–0.595	<0.001
Lymph nodes	85.4%	0.524	0.437–0.611	<0.001
ACR-TIRADS	60.0%	0.466	0.405–0.528	<0.001
EU-TIRADS	58.41%	0.368	0.291–0.444	<0.001
K-TIRADS	46.0%	0.273	0.209–0.338	<0.001

Abbreviations: CI = Confidence interval, *κ* = kappa value, ACR = American College of Radiology, EU = European Thyroid Association, K = Korean Thyroid Association. * Fleiss kappa analysis.

**Table 4:** Interobserver agreement for I-TIRADS parameters and established RSSs in the Intermediate group.

Parameters	Interobserver Agreement (%)	*κ*	95% CI	*p*-Value *
Composition	70.2%	0.464	0.387–0.540	<0.001
Echogenicity	61.3%	0.429	0.358–0.500	<0.001
Margin	66.7%	0.342	0.258–0.427	<0.001
Direction of growth	89.2%	0.491	0.381–0.602	<0.001
Echogenic foci/calcifications	54.9%	0.393	0.330–0.457	<0.001
Extrathyroidal extension	71.75%	0.185	0.098–0.272	<0.001
Lymph nodes	80.0%	0.227	0.139–0.316	<0.001
ACR-TIRADS	42.2%	0.217	0.155–0.279	<0.001
EU-TIRADS	40.0%	0.133	0.062–0.204	<0.001
K-TIRADS	36.83%	0.139	0.074–0.204	<0.001

Abbreviations: CI = Confidence interval, *κ* = kappa value, ACR = American College of Radiology, EU = European Thyroid Association, K = Korean Thyroid Association. * Fleiss kappa analysis.

**Table 5:** Interobserver agreement for I-TIRADS parameters and established RSSs in the Junior group.

Parameters	Interobserver Agreement (%)	*κ*	95% CI	*p*-Value *
Composition	65.4%	0.403	0.325–0.481	<0.001
Echogenicity	51.8%	0.220	0.148–0.307	<0.001
Margin	46.0%	0.076	−0.009–0.160	0.078
Direction of growth	87.9%	0.431	0.321–0.542	<0.001
Echogenic foci/calcifications	47.6%	0.252	0.183–0.320	<0.001
Extrathyroidal extension	70.1%	0.194	0.109–0.278	<0.001
Lymph nodes	68.3%	0.118	0.031–0.204	<0.001
ACR-TIRADS	39.3%	0.134	0.066–0.202	<0.001
EU-TIRADS	48.6%	0.233	0.159–0.308	<0.001
K-TIRADS	37.5%	0.147	0.082–0.212	<0.001

Abbreviations: CI = Confidence interval, *κ* = kappa value, ACR = American College of Radiology, EU = European Thyroid Association, K = Korean Thyroid Association. * Fleiss kappa analysis.

## Data Availability

The data supporting the findings of this study are not publicly available due to privacy and ethical restrictions. However, the data may be available from the corresponding author upon reasonable request and subject to institutional approval.

## Supplementary Materials

The following supporting information can be downloaded at: https://www.mdpi.com/article/10.3390/diagnostics16193103/s1; Table S1: Baseline characteristics of sex, nodule, and pathology outcomes; Table S2: Interobserver agreement (*κ*) levels in subgroups according to I-TIRADS parameters and established RSSs within the Senior group; Table S3: Interobserver agreement (*κ*) levels in subgroups according to I-TIRADS parameters and established RSSs within the Intermediate group; Table S4: Interobserver agreement (*κ*) levels in subgroups according to I-TIRADS parameters and established RSSs within the Junior group.

## Abbreviations

ACR-TIRADS	American College of Radiology Thyroid Imaging Reporting and Data System
AI	Artificial intelligence
BSRTC	Bethesda System for Reporting Thyroid Cytopathology
CI	Confidence Interval
ETE	Extrathyroidal Extension
EU-TIRADS	European Thyroid Imaging Reporting and Data System
FNAB	Fine-needle aspiration biopsy
I-TIRADS	International Thyroid Imaging Reporting and Data System
K-TIRADS	Korean Thyroid Imaging Reporting and Data System
PTC	Papillary Thyroid Carcinoma
ROC AUC	Receiver Operating Characteristic Area Under the Curve
RSSs	Risk stratification systems
SD	Standard Deviation
SPSS	Statistical Package for the Social Sciences
US	Ultrasonography
