# Supplementary material for: Accelerating the Radiology Learning Curve: How the Parameter-Based I-TIRADS Lexicon Enhances Interpretive Reliability and Learning Curves Across Resident Experience Levels—A Dynamic Video-Based Study
Source: Diagnostics (Basel). 2026 Sep 24;16(19):3103. doi: 10.3390/diagnostics16193103 (PMC13664508; doi:10.3390/diagnostics16193103)
Supplement: Supplementary file 1 [file diagnostics-16-03103-s001.zip › diagnostics-4508839-supplementary.pdf]

Supplementary Tables

Table S1. Baseline characteristics of sex, nodule, and pathology outcomes

**Parameters**		**Number (n)**	**Frequency (%)**
**Sex**	Male	16	15.2%
	Female	89	84.8%
**Nodule multiplicity**	Solitary	14	13.3%
	Multiple	91	86.7%
**Nodule size distribution**	<1 cm	7	6.7%
	≥1 cm	98	93.3%
**Bethesda classification**	Bethesda I	0	0.0%
	Bethesda II	64	61.0%
	Bethesda III	31	29.5%
	Bethesda IV	1	1.0%
	Bethesda V	1	1.0%
	Bethesda VI	8	7.6%

Table S2. Interobserver agreement (κ) levels in subgroups according to I-TIRADS parameters and established RSSs within the Senior group

**Parameters**	**Subgroup**	***k***	**95% CI**		**P** *
			**Lower limit**	**Upper limit**	
**Composition**	Solid	0.757	0.647	0.867	**<0.001**
	Mixed, predominantly solid	0.592	0.481	0.702	**<0.001**
	Mixed, predominantly cystic	0.720	0.610	0.830	**<0.001**
	Spongiform	0.897	0.787	1.007	**<0.001**
	Pure cyst	–	–	–	–
**Echogenicity**	Markedly hypoechoic	0.618	0.508	0.729	**<0.001**
	Mildly hypoechoic	0.509	0.398	0.619	**<0.001**
	Isoechoic	0.646	0.536	0.757	**<0.001**
	Hyperechoic	0.694	0.583	0.804	**<0.001**
	Anechoic	0.497	0.386	0.607	**<0.001**
**Margin**	Irregular	0.786	0.676	0.897	**<0.001**
	Ill-defined	0.626	0.515	0.736	**<0.001**
	Smooth	0.741	0.630	0.851	**<0.001**
**Direction of growth**	Taller-than-wide (TTW)	0.826	0.716	0.937	**<0.001**
	Wider-than-tall (WTT)	0.826	0.716	0.937	**<0.001**
**Echogenic foci/calcifications**	Punctate echogenic foci/microcalcifications	0.786	0.676	0.896	**<0.001**
	Macrocalcifications	0.707	0.597	0.818	**<0.001**
	Peripheral (rim) calcifications	0.660	0.550	0.771	**<0.001**
	Echogenic foci with comet-tail artifact	0.833	0.723	0.944	**<0.001**
	No calcification	0.864	0.753	0.974	**<0.001**
**Extrathyroidal extension**	Gross extrathyroidal extension	0.529	0.419	0.639	**<0.001**
	Suspicious minor extrathyroidal extension	0.409	0.298	0.519	**<0.001**
	Capsule contact	0.569	0.459	0.680	**<0.001**
**Lymph nodes**	Suspicious lymph node	0.680	0.569	0.790	**<0.001**
	Indeterminate lymph node	0.435	0.325	0.546	**<0.001**
	Non-suspicious lymph node	0.522	0.412	0.633	**<0.001**
**ACR-TIRADS**	1	0.796	0.685	0.906	**<0.001**
	2	0.521	0.411	0.632	**<0.001**
	3	0.443	0.333	0.554	**<0.001**
	4	0.393 [b]	0.282	0.503	**<0.001**
	5	0.429	0.319	0.540	**<0.001**
**EU-TIRADS**	2	0.187 [b]	0.077	0.298	**0.001**
	3	0.459	0.349	0.570	**<0.001**
	4	0.190 [b]	0.080	0.301	**0.001**
	5	0.448	0.338	0.558	**<0.001**
**K-TIRADS**	2	0.243 [b]	0.132	0.353	**<0.001**
	3	0.278 [b]	0.168	0.389	**<0.001**
	4	0.191 [b]	0.081	0.302	**0.001**
	5	0.422	0.311	0.532	**<0.001**

Abbreviations: CI = Confidence interval, k = kappa value, ACR = American College of Radiology, EU = European Thyroid Association, K = Korean Thyroid Association.

[b] *Very poor or fair consensus (k <0.40; p<0.05) are marked.*

**Fleiss kappa analysis.*

Table S3. Interobserver agreement (κ) levels in subgroups according to I-TIRADS parameters and established RSSs within the Intermediate group

Parameters	Subgroup	*k*	95% CI		P *
			Lower limit	Upper limit	
**Composition**	Solid	0.557	0.447	0.668	**<0.001**
	Mixed, predominantly solid	0.280 [b]	0.169	0.390	**<0.001**
	Mixed, predominantly cystic	0.567	0.456	0.677	**<0.001**
	Spongiform	0.505	0.395	0.616	**<0.001**
	Pure cyst	–	–	–	–
**Echogenicity**	Markedly hypoechoic	0.600	0.489	0.710	**<0.001**
	Mildly hypoechoic	0.357 [b]	0.247	0.468	**<0.001**
	Isoechoic	0.463	0.353	0.573	**<0.001**
	Hyperechoic	0.271 [b]	0.160	0.381	**<0.001**
	Anechoic	-0.003 [a]	-0.114	0.107	0.955
**Margin**	Irregular	0.282 [b]	0.171	0.392	**<0.001**
	Ill-defined	0.218 [b]	0.108	0.329	**<0.001**
	Smooth	0.467	0.356	0.577	**<0.001**
**Direction of growth**	Taller-than-wide (TTW)	0.491	0.381	0.602	**<0.001**
	Wider-than-tall (WTT)	0.491	0.381	0.602	**<0.001**
**Echogenic foci/calcifications**	Punctate echogenic foci/microcalcifications	0.400	0.290	0.510	**<0.001**
	Macrocalcifications	0.406	0.295	0.516	**<0.001**
	Peripheral (rim) calcifications	0.150 [b]	0.040	0.261	**0.008**
	Echogenic foci with comet-tail artifact	0.414	0.304	0.525	**<0.001**
	No calcification	0.378 [b]	0.268	0.489	**<0.001**
**Extrathyroidal extension**	Gross extrathyroidal extension	0.267 [b]	0.157	0.377	**<0.001**
	Suspicious minor extrathyroidal extension	0.057 [a]	-0.053	0.168	0.309
	Capsule contact	0.244 [b]	0.134	0.354	**<0.001**
**Lymph nodes**	Suspicious lymph node	0.300 [b]	0.190	0.410	**<0.001**
	Indeterminate lymph node	0.177 [b]	0.067	0.288	**0.002**
	Non-suspicious lymph node	0.236 [b]	0.126	0.347	**<0.001**
**ACR-TIRADS**	1	0.414	0.303	0.524	**<0.001**
	2	0.132 [b]	0.022	0.243	**0.019**
	3	0.151 [b]	0.040	0.261	**0.007**
	4	0.175 [b]	0.065	0.286	**0.002**
	5	0.358 [b]	0.248	0.469	**<0.001**
**EU-TIRADS**	2	-0.014 [a]	-0.125	0.096	0.798
	3	0.140 [b]	0.030	0.251	**0.013**
	4	0.051 [a]	-0.059	0.162	0.909
	5	0.248 [b]	0.138	0.359	**<0.001**
**K-TIRADS**	2	0.118 [b]	0.007	0.228	**0.037**
	3	0.180 [b]	0.069	0.290	**0.001**
	4	0.091 [a]	-0.019	0.202	0.105
	5	0.173 [b]	0.063	0.283	**0.002**

Abbreviations: CI = Confidence interval, k = kappa value, ACR = American College of Radiology, EU = European Thyroid Association, K = Korean Thyroid Association.
[a] *No significant consensus was observed (K<0.20; p>0.05).*
[b] *Very poor or fair consensus (k <0.40; p<0.05) are marked.*
[*] *Fleiss kappa analysis.*

Table S4. Interobserver agreement (κ) levels in subgroups according to I-TIRADS parameters and established RSSs within the Junior group

Parameters	Subgroup	*k*	95% CI		P *
			Lower limit	Upper limit	
**Composition**	Solid	0.531	0.420	0.641	**<0.001**
	Mixed, predominantly solid	0.362 [b]	0.252	0.472	**<0.001**
	Mixed, predominantly cystic	0.210 [b]	0.099	0.320	**<0.001**
	Spongiform	0.255 [b]	0.145	0.366	**<0.001**
	Pure cyst	–	–	–	–
**Echogenicity**	Markedly hypoechoic	0.261 [b]	0.151	0.372	**<0.001**
	Mildly hypoechoic	0.199 [b]	0.089	0.310	**<0.001**
	Isoechoic	0.280 [b]	0.170	0.390	**<0.001**
	Hyperechoic	0.104 [a]	-0.007	0.214	0.065
	Anechoic	-0.006 [a]	-0.117	0.104	0.910
**Margin**	Irregular	0.192 [b]	0.082	0.302	**0.001**
	Ill-defined	-0.006 [a]	-0.116	0.105	0.921
	Smooth	0.092 [a]	-0.019	0.202	0.103
**Direction of growth**	Taller-than-wide (TTW)	0.431	0.321	0.542	**<0.001**
	Wider-than-tall (WTT)	0.431	0.321	0.542	**<0.001**
**Echogenic foci/calcifications**	Punctate echogenic foci/microcalcifications	0.243 [b]	0.132	0.353	**<0.001**
	Macrocalcifications	0.391 [b]	0.280	0.501	**<0.001**
	Peripheral (rim) calcifications	-0.003 [a]	-0.114	0.107	0.955
	Echogenic foci with comet-tail artifact	-0.057 [a]	-0.168	0.053	0.309
	No calcification	0.396 [b]	0.286	0.507	**<0.001**
**Extrathyroidal extension**	Gross extrathyroidal extension	0.284 [b]	0.174	0.395	**<0.001**
	Suspicious minor extrathyroidal extension	0.093 [a]	-0.018	0.203	0.10
	Capsule contact	0.210 [b]	0.100	0.321	**<0.001**
**Lymph nodes**	Suspicious lymph node	0.188 [b]	0.078	0.299	**0.001**
	Indeterminate lymph node	0.075 [a]	-0.035	0.186	0.181
	Non-suspicious lymph node	0.119 [b]	0.008	0.229	**0.035**
**ACR-TIRADS**	1	0.070 [a]	-0.040	0.181	0.211
	2	0.057 [a]	-0.053	0.168	0.310
	3	0.138 [b]	0.028	0.249	**0.014**
	4	0.033 [a]	-0.077	0.143	0.559
	5	0.295 [b]	0.185	0.406	**<0.001**
**EU-TIRADS**	2	0.058 [a]	-0.052	0.168	0.303
	3	0.267 [b]	0.157	0.378	**<0.001**
	4	0.146 [b]	0.036	0.257	**0.009**
	5	0.288 [b]	0.177	0.398	**<0.001**
**K-TIRADS**	2	-0.032 [a]	-0.143	0.078	0.566
	3	0.199 [b]	0.089	0.310	**<0.001**
	4	0.057 [a]	-0.053	0.168	0.310
	5	0.318 [b]	0.208	0.429	**<0.001**

Abbreviations: CI = Confidence interval, k = kappa value, ACR = American College of Radiology, EU = European Thyroid Association, K = Korean Thyroid Association.
[a] *No significant consensus was observed (K<0.20; p>0.05).*
[b] *Very poor or fair consensus (k <0.40; p<0.05) are marked.*
[*] *Fleiss kappa analysis*
